# Predicting the regrowth of clinically non-functioning pituitary adenoma with a statistical model

**DOI:** 10.1186/s12967-019-1915-2

**Published:** 2019-05-20

**Authors:** Sen Cheng, Jiaqi Wu, Chuzhong Li, Yangfang Li, Chunhui Liu, Guilin Li, Wuju Li, Shuofeng Hu, Xiaomin Ying, Yazhuo Zhang

**Affiliations:** 1 Beijing Neurosurgical Institute, Capital Medical University, Beijing, 100070 People’s Republic of China; 2 Institute of Military Cognition and Brain Sciences, Academy of Military Medical Sciences, No. 27, Taiping Road, Haidian District, Beijing, 100850 People’s Republic of China; 3 Neurosurgery, Beijing Neurosurgical Institute, Beijing Tiantan Hospital Affiliated To Capital Medical University, Beijing Institute for Brain Disorders Brain Tumour Center, China National Clinical Research Center for Neurological Diseases, Key Laboratory of Central Nervous System Injury Research, Beijing, 100070 People’s Republic of China

**Keywords:** Clinically non-functioning pituitary adenoma, Regrowth, Predicting model

## Abstract

**Background:**

Compared with clinically functioning pituitary adenoma (FPA), clinically non-functioning pituitary adenoma (NFPA) lacks of detectable hypersecreting serum hormones and related symptoms which make it difficult to predict the prognosis and monitoring for postoperative tumour regrowth. We aim to investigate whether the expression of selected tumour-related proteins and clinical features could be used as tumour markers to effectively predict the regrowth of NFPA.

**Method:**

Tumour samples were collected from 295 patients with NFPA from Beijing Tiantan Hospital. The expression levels of 41 tumour-associated proteins were assessed using tissue microarray analyses. Clinical characteristics were analysed via univariate and multivariate logistic regression analyses. Logistic regression algorithm was applied to build a prediction model based on the expression levels of selected proteins and clinical signatures, which was then assessed in the testing set.

**Results:**

Three proteins and two clinical signatures were confirmed to be significantly related to the regrowth of NFPA, including cyclin-dependent kinase inhibitor 2A (CDKN2A/p16), WNT inhibitory factor 1 (WIF1), tumour growth factor beta (TGF-β), age and tumour volume. A prediction model was generated on the training set, which achieved a fivefold predictive accuracy of 81.2%. The prediction ability was validated on the testing set with an accuracy of 83.9%. The area under the receiver operating characteristic curves (AUC) for the signatures were 0.895 and 0.881 in the training and testing sets, respectively.

**Conclusion:**

The prediction model could effectively predict the regrowth of NFPA, which may facilitate the prognostic evaluation and guide early interventions.

**Electronic supplementary material:**

The online version of this article (10.1186/s12967-019-1915-2) contains supplementary material, which is available to authorized users.

## Background

Pituitary adenoma (PA) constitutes approximately 15% of all intracranial neoplasm [1]. NFPA is a special type of pituitary adenoma that generally shows no clinical symptoms such as serum hormone level elevation apart from mass effect, including visual disturbance, headache and various degree of hypopituitarism [2, 3]. Surgery is the first choice of treatment for most patients and no effective drugs can ameliorate its prognosis. However, about 12–58% of patients with macro-adenoma will experience tumour regrowth within 5 years even the tumour remnants was undetectable during surgery or on post-operation magnetic resonance imaging (MRI) [4]. However, we cannot currently predict which patients will experience tumour regrowth after surgery. Although radiotherapy is effective for the reduction of recurrence, the potential risk for hypopituitarism or optic nerve injury limits its application. Therefore, clinical or pathological parameters for predicting such regrowth behavior are of necessity.

Few systematic reports have discussed the prediction of the regrowth possibility of NFPA using statistical prediction models. In this study, we identified 41 key proteins whose expression were related to tumorigenesis and progression of pituitary adenoma. We then established a regrowth prediction model with two clinical signatures and three tumour-associated proteins whose expression levels were varied in NFPA. The main purpose of our study was to determine whether we could build a prediction model and validate its authenticity. Our study may facilitate the prognostic evaluation and early intervention to patients with NFPA.

## Materials and methods

### Patients selection

We retrospectively collected regrowth and non-regrowth tissue from 295 patients diagnosed with NFPA at Beijing Tiantan Hospital from April 2006 to September 2014. All patients were performed enhanced head MRI scan before and after surgery in order to assess tumour volume, Knosp classification and tumour resection grade. The minimum follow-up time was 36 months, and the average follow-up time was 86.5 months (range 36–137 months). Regrowth of NFPA was defined as a tumour maximum diameter increases from any direction on MRI of more than 2 mm from the day of surgery to follow-up endpoint with or without the reappearance of visual disturbance, headache or hypopituitarism.

### Sample preparation

NFPA samples were stored and fixed in 10% neutral-buffered formalin immediately upon removal from the sellar region and then embedded in paraffin. Specimen slides stained with hematoxylin and eosin (H&E) were viewed by two different pathologists, who marked the tumour tissues on the samples.

### Tissue microarray analyses

Two 2.0-mm diameter samples were removed then transferred to a recipient paraffin block to construct TMAs using a Tissue Array MiniCore 3 (ALPHELYS, Plaisir, France). Samples (4-µm thick) were obtained from each TMA with Leica Rotary Microtome RM2135 (Wetzlar, Germany). Immunohistochemical staining was performed on Leica BOND III automated system. All samples were pretreated in a 65 °C oven for 1.5 h. Staining was performed with dewaxing and epitope retrieval. Samples were blocked with peroxide solution for 7 min. Selected antibodies (shown in Additional file 1: Table S1) were used to incubate samples, followed by post-primary for 8 min, polymer for 8 min, diaminobenzidine (DAB) for 5 min, and hematoxylin counterstain for 2 min. Finally, the samples were dehydrated, cleared, then fixed with neutral resins. Stained samples were scanned with Leica Aperio AT2 scanner (400× magnification) and analysed by two different pathologists. Samples were scored as negative (0+), weak (1+), moderate (2+), and strong (3+) signal. The percentage of positivity was also calculated by two pathologists. The histological scores (H-Score) was calculated using following formula: H-Score = 0 × (percentage of negative) + 1 × (percentage of weak) + 2 × (percentage of moderate) + 3 × (percentage of strong). Thus, the H-Score ranges from 0 to 300 [5].

### Statistical analyses

**Fig. 1:**
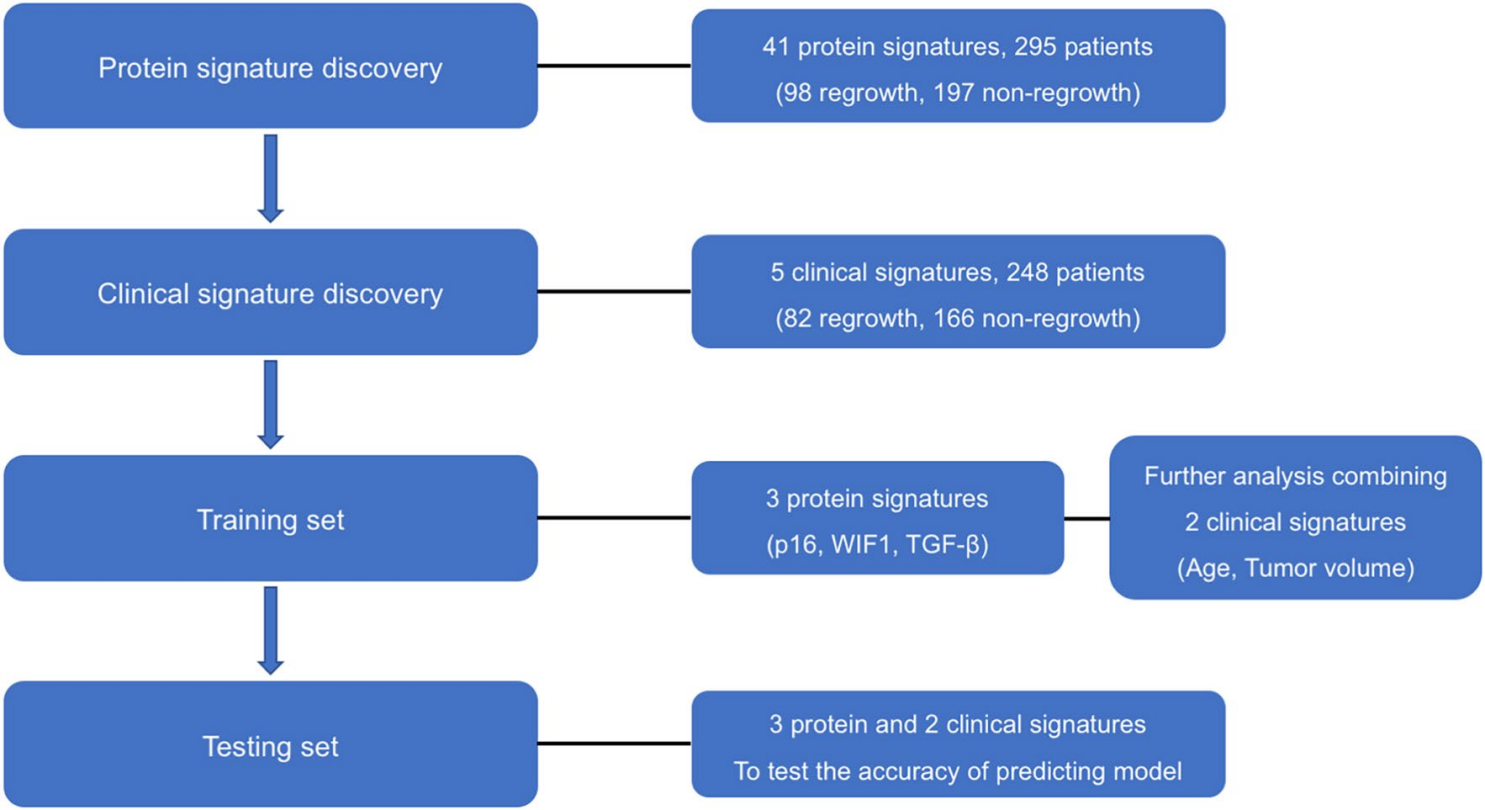
Flowchart of the study

The flowchart of the study is shown in Fig. 1. For candidate protein signatures analyses, the differences in expression levels were evaluated with Wilcoxon–Mann–Whitney test. Kaplan–Meier analyses with two-sided log-rank test was used to determine the significance of the survival differences between the two groups, and hazard ratio (HR) was calculated using Cox proportional hazards regression model. The clinical characteristics were analysed with univariate and multivariate logistic regression analyses. Logistic regression algorithm was employed to develop the classification model. The prediction performance of the prediction model was evaluated in terms of the discriminatory accuracy in the training and testing set respectively. The discrimination performance of biomarkers for predicting the regrowth of NFPA was assessed by generating receiver operating characteristic (ROC) curve, the area under the ROC curve (AUC) was used to evaluate the classification performance. All statistical analyses and modeling were performed in R (version 3.1) and IBM SPSS Statistics for Windows, version 21.0. The differences were considered to be statistically significant for *P* < 0.05.

## Results

### Discovering candidate protein signatures in tumour tissues

**Fig. 2:**
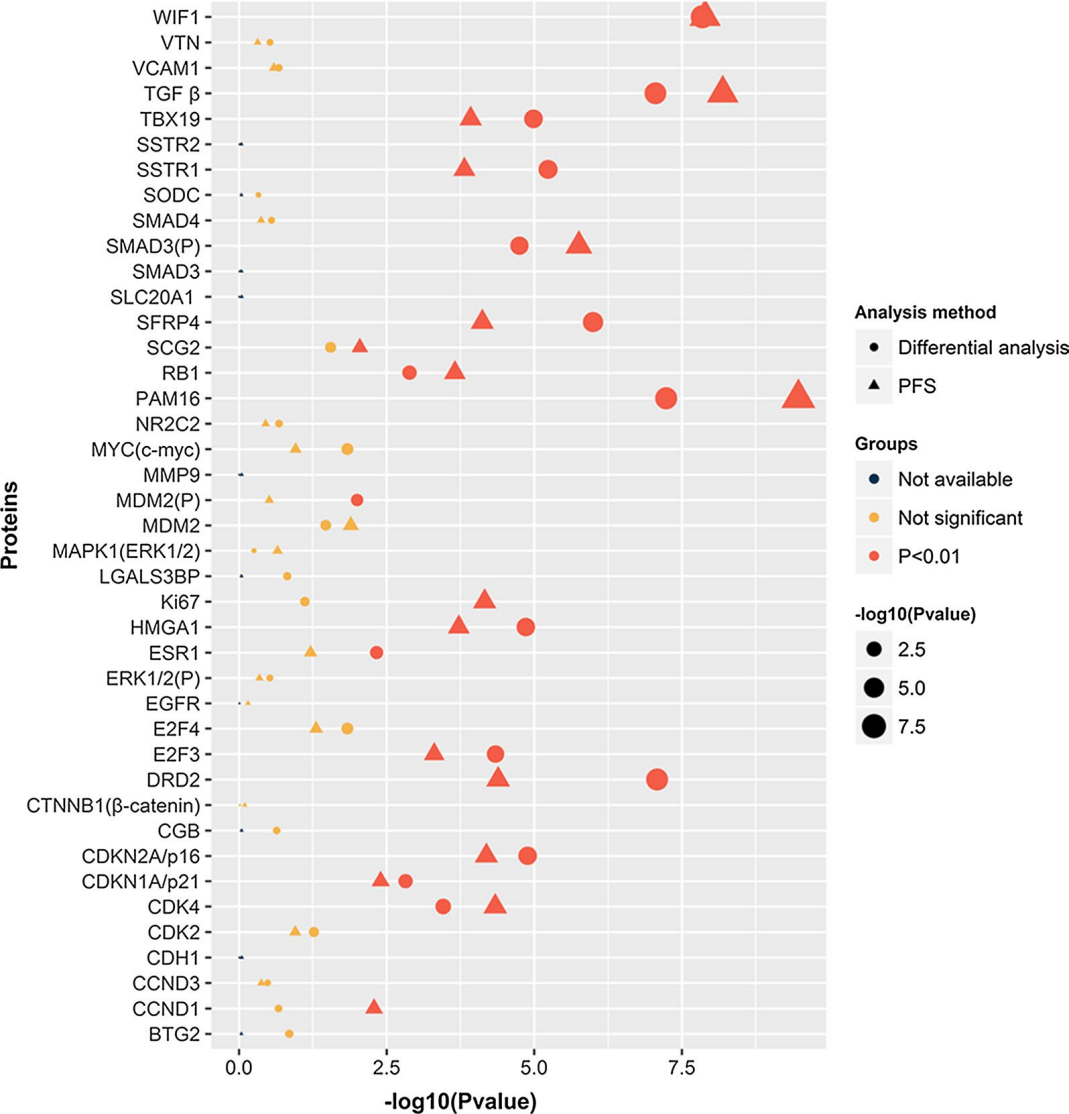
Differential and Kaplan–Meier analyses of protein signatures. Scatter diagram showing the statistical significance results of 41 tumour related proteins. Sixteen and seventeen protein signatures were valuable in differential and Kaplan–Meier analyses, respectively (P < 0.01)

**Fig. 3:**
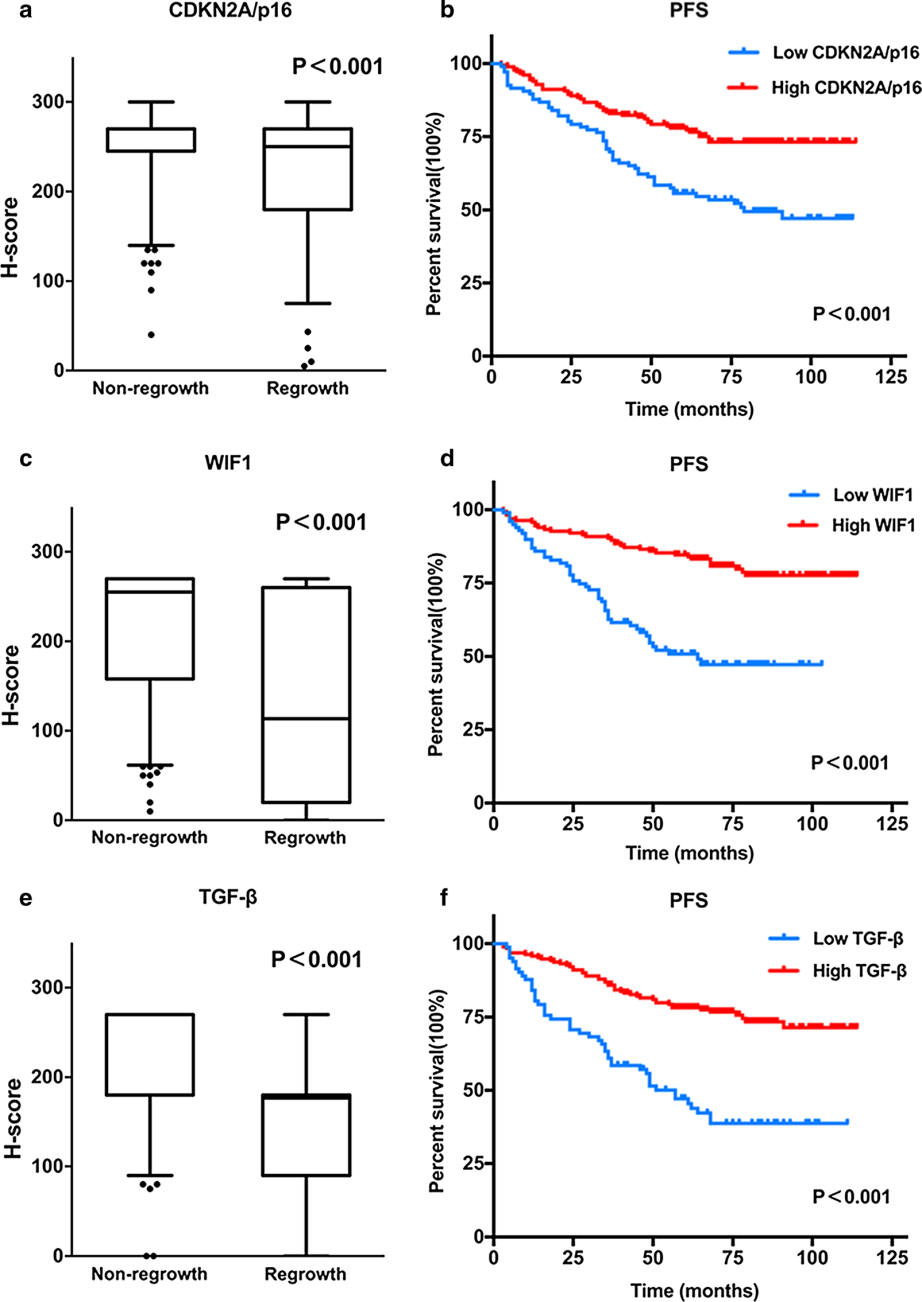
Differential and Kaplan–Meier analyses of the three protein signatures in patients with NFPA. Distributions of p16, WIF1 and TGF-β protein expression levels in patients of non-regrowth and regrowth cohorts (**a**, **c**, **e**). The Kaplan–Meier curves of PFS between the low and high median protein expression level groups are also shown (**b**, **d**, **f**)

Tumour tissues from 295 cases including 98 regrowth patients and 197 non-regrowth patients were entered into this study. The expression levels of 41 tumour-related proteins in regrowth and non-regrowth tumour were determined via microarray analyses. We then compared the differential expression levels between the two groups, and selected 16 different proteins (presequence translocase-associated motor 16 [PAM16], TGF-β, WIF1, mothers against decapentaplegic homolog 3 [SMAD3(P)], Dopamine receptor D_2_ [DRD2], cyclin-dependent kinase 4 [CDK4], CDKN2A/p16, secreted frizzled-related protein 4 [SFRP4], T-box transcription factor [TBX19], somatostatin receptor 1 [SSTR1], high mobility group AT-hook 1 [HMGA1], retinoblastoma protein [RB1], E2F3, cyclin-dependent kinase inhibitor 1 [CDKN1A/p21], estrogen receptor 1 [ESR1], and mouse double minute 2 homolog [MDM2]) that were valuable (*P* < 0.01, Fig. 2). Meanwhile, in order to assess the associations between protein expression levels with regrowth, we performed Kaplan–Meier analyses to further identify whether there are possible candidates. The event endpoint was calculated from surgical resection to the date of the first regrowth. Patients are divided into 2 groups, low-expression group and high-expression group, according to the median cut-off of the protein expression levels. 17 proteins (PAM16, TGF-β, WIF1, SMAD3(P), DRD2, CDK4, CDKN2A/p16, Ki67, SFRP4, TBX19, SSTR1, HMGA1, RB1, E2F3, CDKN1A/p21, cyclin-D1 [CCND1], and secretogranin II [SCG2]) were valuable (*P* < 0.01) including the previous 14 proteins (Fig. 3). Therefore, we used the intersection of the signatures of these 14 proteins to perform further investigations. There are 203 cases expressed all 14 proteins without missing values.

### Protein signatures analyses

Based on the 14 candidate protein signatures from the intersection of significant proteins in the differential analyses and survival analyses, a logistic regression analysis was used to screen the most likely candidate factors for tumour regrowth (shown in Additional file 2: Table S2). Finally, three proteins, WIF1, p16 and TGF-β, with the p values less than 0.05 were selected as predictors for the further establishment of the prediction model (Fig. 3). There are 248 cases expressed all three proteins without missing values; therefore, we chose 248 cases to perform the clinical characteristic analyses and the establishment of the prediction model. The 248 patients were randomly grouped into training and testing sets in a 3:1 ratio using computer-generated random numbers.

### Patients characteristics

We preliminarily selected sex, age (< 40 years as grade 0, 40–60 years as grade 1, and ≥ 60 years as grade 2), tumour volume (macro-adenoma: 10–40 mm as grade 1, and giant adenoma: > 40 mm as grade 2), tumour resection rate (total resection as grade 1, subtotal resection as grade 2, partial resection as grade 3) and Knosp grade (0, 1, 2, 3, and 4) as candidate clinical predictors for the prediction of tumour regrowth. All of the selected clinical characteristics were categorical variables. The clinical factors that were statistically significantly associated with the regrowth of NFPA are shown in Table 1. Univariate logistic regression analyses showed that gender was not related to tumour regrowth (*P* = 0.067). Age, Knosp grade, tumour volume and tumour resection rate were found to be correlated with regrowth (*P* < 0.05).

**Table 1:** Clinical characteristics of the patients with NFPA

Characteristics	All patients (n = 295)	Regrowth cohort (n = 98)	Non-regrowth cohort (n = 197)	Univariate analysis		Multivariate analysis	
				OR (95% CI)	p value	OR (95% CI)	p value
Gender					0.067		–
Female	140 (47.46%)	56 (57.14%)	84 (42.64%)	1.00 (referent)		–	
Male	155 (52.54%)	42 (42.86%)	113 (57.36%)	0.61 (0.36 to 1.04)		–	
Age					< 0.001		< 0.001
< 40	61 (20.68%)	38 (38.78%)	23 (11.68%)	1.00 (referent)		1.00 (referent)	
40–60	177 (60.00%)	52 (53.06%)	125 (63.45%)	0.20 (0.10 to 0.39)		0.22 (0.11 to 0.43)	
≥ 60	57 (19.32%)	8 (8.16%)	49 (24.87%)	0.09 (0.03 to 0.24)		0.11 (0.04 to 0.30)	
Tumour volume					< 0.001		0.01
Macro	181 (61.36%)	46 (46.94%)	135 (68.53%)	1.00 (referent)		1.00 (referent)	
Giant	114 (38.64%)	52 (53.06%)	62 (31.47%)	2.68 (1.55 to 4.62)		2.15 (1.20 to 3.87)	
Knosp grade					0.022		0.349
0	62 (21.02%)	11 (11.22%)	51 (25.89%)	1.00 (referent)		–	
1	61 (20.68%)	21 (21.43%)	40 (20.31%)	1.71 (0.63 to 4.67)		–	
2	41 (13.90%)	13 (13.27%)	28 (14.21%)	2.31 (0.83 to 6.41)		–	
3	27 (9.15%)	12 (12.24%)	15 (7.61%)	4.29 (1.45 to 12.65)		–	
4	104 (35.25%)	41 (41.84%)	63 (31.98%)	3.56 (1.49 to 8.48)		–	
Tumour resection rate					0.003		0.254
Total	143 (48.47%)	38 (38.78%)	105 (53.30%)	1.00 (referent)		–	
Subtotal	60 (20.34%)	16 (16.32%)	44 (22.33%)	1.15 (0.55 to 2.42)		–	
Partial	92 (31.19%)	44 (44.90%)	48 (24.37%)	2.75 (1.50 to 5.04)		–	

OR, odds ratios; 95% CI, 95% confidence intervals; Micro, micro-adenoma; Macro, macro-adenoma; Giant, giant adenoma; Total, total resection; Subtotal, subtotal resection; Partial, partial resection

### Clinical signature analyses

To further determine the underlying significance of the selected clinical signatures, multivariate logistic regression analyses was performed to identify independent factors that were statistically significantly associated with the regrowth of NFPA patients. Age (*P* < 0.01) and tumour volume (*P* = 0.01) were independent risk factors of regrowth (Table 1). Our analyses indicated that the regrowth inclination decreases with age, and patients younger than 40 years are more prone to regrowth compared with those aged from 40 to 60 years (OR = 0.2, 95% CI = 0.10 to 0.39). In addition, patients aged over 60 years are least likely to have tumour regrowth (OR = 0.09, 95% CI = 0.03 to 0.24). The tumour volume has a positive correlation with the risk of tumour regrowth. Compared with patients with macro-adenoma, patients with giant adenoma were more inclined to suffer tumour regrowth (OR = 2.68, 95% CI = 1.55 to 4.62).

### Establishment of the regrowth prediction model

Based on the clinical and protein signatures, we established a regrowth prediction model using logistic regression algorithm in the training set, the final prediction model was expressed by the following equation

Y = − 0.0159 × p16 − 0.0067 × WIF1 − 0.0162 × TGF-β − 1.2260 × age grade + 1.2205 × gross tumor volume grade + 6.4351.

The discriminant score P was determined as: P = exp(Y)/(1 + exp(Y)). In our classification model, the cut-off score was set at 0.5. When the discriminant score P was calculated to be less than 0.5, the patient was classified as a non-regrowth case, the case was predicted to be a regrowth.

**Fig. 4:**
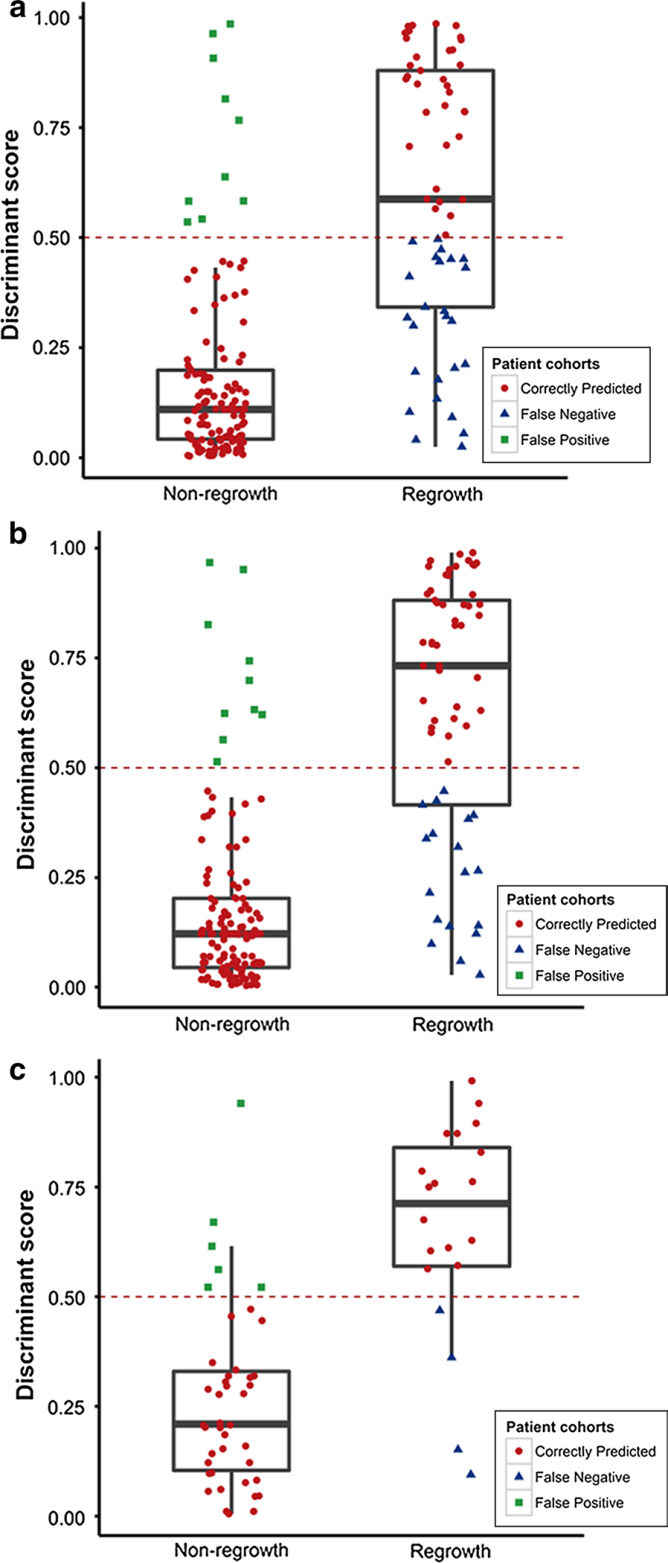
Distributions of the discriminant scores predicted by the model for the different groups. Box-and-whisker plot showing the distributions of the discriminant scores of the non-regrowth and regrowth groups in the overall fivefold cross-validation cases (**a**), the training (**b**) and testing set (**c**). The three different shapes (or colours) in the boxplot respectively indicates the patients who were correctly predicted and mis-predicted (false positive and false negative)

During the training phase, a fivefold cross-validation accuracy was used to assess the unbiased performance of the logistic regression-based classifier in distinguishing regrowth and non-regrowth patients. For the training set of 186 fivefold cross-validation cases, the combined signature provided an overall accuracy of 81.2% (151 of 186), in which 36 of 61 regrowth cases and 115 of 125 non-regrowth cases were correctly predicted (Fig. 4a). The final classification model has obtained a better prediction accuracy in the training set, which provided an accuracy of 84.9%. In this set, 158 of 186 patients were correctly classified, of the 28 patients misclassified, 18 were false-negative and 10 were false-positive, respectively (Fig. 4b). Meanwhile, the prediction ability was validated in the testing set, with an accuracy of 83.9% (52 of 62). Correct predictions were obtained for 16 of 20 (four false-negative cases) regrowth cases and 36 of 42 (six false-positive cases) non-regrowth cases (Fig. 4c).

**Fig. 5:**
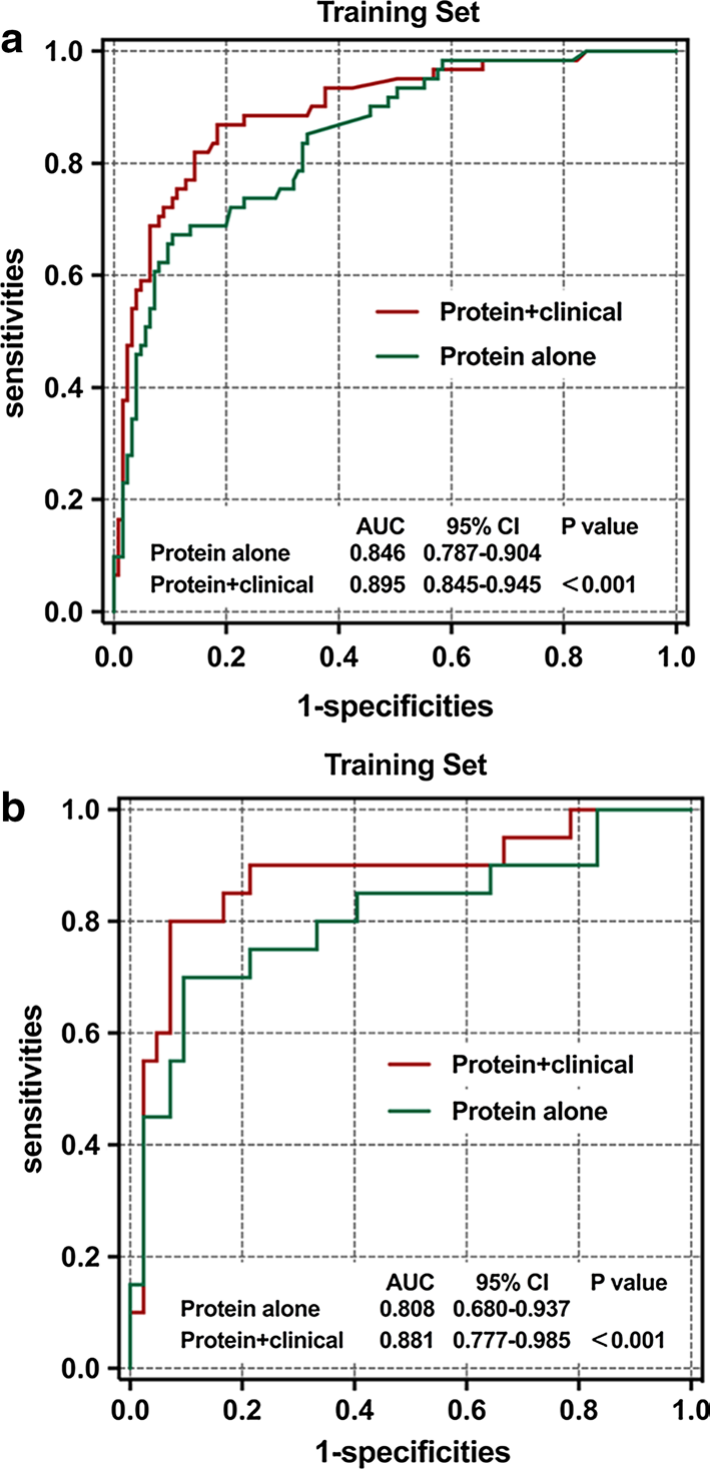
The regrowth prediction model in the training and testing sets. Two ROC curves showing the comparisons of the sensitivity and specificity for the prediction of regrowth in protein combining clinical signature and protein signature alone in the training (**a**) and testing set (**b**). The combination of protein and clinical signatures shows a better prediction accuracy than that of protein signature alone (P < 0.001)

The ROC analyses were used to assess the sensitivity and specificity of the regrowth prediction. Our classification model based on the combination of clinical and protein signatures showed a good sensitivity and specificity for regrowth prediction with an AUC of 0.895 in the training set (Fig. 5a). We thus validated the model using the same discriminant score in the testing set. The predictive ability was remarkably stable with an AUC of 0.881. In order to explore the possibility whether proteins combined with clinical signatures could be better biomarkers for regrowth prediction, we constructed a model using the protein signatures alone and compared its predictive ability with the model integrating the protein and clinical signatures. The combined model had a significantly better predictive ability than that of the protein signature-alone model, in both the training set (AUC: 0.895 to 0.846, *P* < 0.001, Fig. 5a) and testing set (AUC: 0.881 to 0.808, *P* < 0.001, Fig. 5b). The introduction of clinical signatures performed remarkably well.

## Discussion

As a benign tumour derived from the anterior pituitary gland, some pituitary adenomas, like FPA, can induce endocrine disorders by the hypersecretion of hormone, such as adrenocorticotropic hormone (ACTH), thyroid-stimulating hormone (TSH), growth hormone (GH) and prolactin (PRL) [6]. The serum hormone levels and corresponding endocrine symptoms provides a feasible approach to qualitative diagnosis and regrowth evaluation. Compared with FPA, NFPA lacks endocrine symptoms such as hormone hypersecretion and is mostly diagnosed and postoperatively monitored via imaging examinations, such as enhanced MRI and computed tomography. Besides the imaging techniques, there are few convenient detection approaches for early diagnosis or regrowth monitoring. The possibility of early intervention and fair prognosis may have vanished when the symptoms of mass effect appear or imaging examination reveals abnormal changes. For these reasons, we dedicate to establish an efficient prediction model for the clinical and pathological practice of NFPA. However, research on molecular markers to predict the regrowth of NFPA is far from comprehensive. Researchers had proposed that some proteins have correlation with NFPA regrowth, but few had minutely illustrated their practical application and none had put forward to aggregate analyses forecast model using these proteins and clinical signatures.

In an attempt to identify proteins that allow us to establish a reliable NFPA regrowth prediction model, we started by screening the key papers of pituitary adenoma to identify 41 tumour related proteins. We then narrowed the set to 14 proteins with differential protein expression and Kaplan–Meier analyses. The final three protein signatures including p16, WIF1 and TGF-β were validated via logistic regression analyses with the p values less than 0.05. They were adopted to build the prediction model in which low expression levels were related to regrowth in our analyses. As far as their biological functions are concerned, low expression level of p16 and WIF1 is observed in pituitary tumorigenesis and is necessary for tumour progression, the methylation of their promoters indicates a poor prognosis [7–11]. The TGF-β signaling pathway is a key player in tumour development whose activity is reportedly lower in NFPA which results in infaust outcomes [12, 13]. The combining effect of these genes leads to the proliferation of tumor cells and the increase of tumor volume.

Previous researchers have taken attempt to predict regrowth with clinical features, but they have not reached a consensus in this respect. Tumour residue has always been considered as a risk factor for regrowth because either trans-sphenoidal surgery or open surgery has difficulties in removing tumour invading cavernous sinus and most patients faced with regrowth in around 5 years [4, 14]. However, in our study, multivariate regression analyses showed that tumour resection rate has no relation with regrowth. This may indicate that regrowth may be associated with the instinct molecular biology characteristic of pituitary adenoma, which also result in the progression of tumour volume, instead of the surgical intervention.

Knosp grade is a pivotal factor in clinical treatment because of its instruction to surgical resection. However, its significance in tumour regrowth is quite controvertible [15–17]. Our research shows that Knosp grade has no influence on tumour regrowth. This may be rational because its potential is revealed by inducing surgical obstacles and directly result in tumour residue which is not an influential factor in our model.

The potential of age as a risk factor for tumour regrowth deserves some consideration. Several studies have reported that younger age to be strongly correlated with regrowth either in relation to residue or as an independent risk factor [16, 18, 19]. However, other researchers hold that age may not show an underlying tendency to regrowth [4, 20]. Our analyses indicates that age is an independent protective factor in patients with NFPA, and younger age indicates a higher regrowth rate.

In recent years, new genetic markers researches including cell-free nucleic acids or long noncoding RNA have been increasingly reported in a variety of tumour, suggesting a promising new class of molecular markers for tumour diagnosis and prognostic evaluation [21–23]. However, these markers have not been fully adapted for clinical use because of their low sensitivity and complicated process. Furthermore, there have been few similar studies in pituitary adenoma except for some research on molecular mechanism [24, 25]. For these reasons, our model combining the clinical features with pathological signatures is a convenient and feasible way to evaluate the postoperative regrowth of NFPA. However, the number of patients in our study was not large enough which restricts us to evaluate the accuracy of our model in an additional validating set.

## Conclusion

To our knowledge, our study is the first to establish a promising statistical model to predict the regrowth of NFPA with clinical and protein signatures. This work provides a novel insight into prognostic evaluation and may help patients to benefit from accurate early intervention.

## Additional files

**Additional file 1: Table S1.** Antibody information involved in the research.

**Additional file 2: Table S2.** P values of 41 tumour related proteins in differential and Kaplan–Meier analyses.

## Data Availability

The datasets during and/or analyzed during the current study available from the corresponding author on reasonable request.

## Abbreviations

PA: pituitary adenoma
FPA: clinically functioning pituitary adenoma
NFPA: clinically non-functioning pituitary adenoma
CDKN2A/p16: cyclin-dependent kinase inhibitor 2A
WIF1: WNT inhibitory factor 1
TGF-β: tumour growth factor beta
ROC: receiver operating characteristic
AUC: area under the receiver operating characteristic curves
